# Glioblastoma Vaccines as Promising Immune-Therapeutics: Challenges and Current Status

**DOI:** 10.3390/vaccines12060655

**Published:** 2024-06-12

**Authors:** Asmae Squalli Houssaini, Salma Lamrabet, Jean Paul Nshizirungu, Nadia Senhaji, Mohammed Sekal, Mehdi Karkouri, Sanae Bennis

**Affiliations:** 1 Laboratory of Biomedical and Translational Research, Faculty of Medicine, Pharmacy and Dental Medicine of Fez, Sidi Mohamed Ben Abdellah University, Fez 30070, Morocco; salma.lamrabet@usmba.ac.ma; 2 Biology Department, School of Science, College of Science and Technology, University of Rwanda, Kigali P.O. Box 3900, Rwanda; j.nshizirungu@ur.ac.rw; 3 Department of Biology, Faculty of Sciences, Moulay Ismail University, Meknes 50000, Morocco; n.senhaji@umi.ac.ma; 4 Laboratory of Epidemiology and Research in Health Sciences, Faculty of Medicine, Pharmacy and Dental Medicine of Fez, Sidi Mohamed Ben Abdellah University, Fez 30070, Morocco; mohammed.sekal@usmba.ac.ma; 5 Department of Pathological Anatomy, Ibn Rochd University Hospital of Casablanca, Casablanca 20250, Morocco; mehdi.karkouri@gmail.com; 6 Laboratory of Cellular and molecular Pathology, Faculty of Medicine and Pharmacy of Casablanca, Hassan II University, Casablanca 20360, Morocco

**Keywords:** glioblastoma, immunotherapy, vaccine, *IDH*, *EGFR*, *TERT*

## Abstract

Glioblastoma (GBM) is the most common and aggressive malignant brain tumor. Standard treatments including surgical resection, radiotherapy, and chemotherapy, have failed to significantly improve the prognosis of glioblastoma patients. Currently, immunotherapeutic approaches based on vaccines, chimeric antigen-receptor T-cells, checkpoint inhibitors, and oncolytic virotherapy are showing promising results in clinical trials. The combination of different immunotherapeutic approaches is proving satisfactory and promising. In view of the challenges of immunotherapy and the resistance of glioblastomas, the treatment of these tumors requires further efforts. In this review, we explore the obstacles that potentially influence the efficacy of the response to immunotherapy and that should be taken into account in clinical trials. This article provides a comprehensive review of vaccine therapy for glioblastoma. In addition, we identify the main biomarkers, including isocitrate dehydrogenase, epidermal growth factor receptor, and telomerase reverse transcriptase, known as potential immunotherapeutic targets in glioblastoma, as well as the current status of clinical trials. This paper also lists proposed solutions to overcome the obstacles facing immunotherapy in glioblastomas.

## 1. Introduction

Glioblastoma is the most aggressive primary brain tumor, requiring greater efforts to improve prognosis. The current standard treatment is based on maximum feasible surgical resection, adjuvant radiotherapy given with concomitant chemotherapy with the DNA alkylating agent temozolomide, and low-intensity alternating electric field therapy (200 Hz) [1]. Alternating electric field therapy is applied via electrodes attached directly to the scalp and interferes with the organelles required for normal cell division. Mitotic disruption ultimately leads to cell cycle arrest and apoptosis [2,3].

Recently, immunotherapy based on vaccines and immune checkpoint inhibitors has been proven remarkably effective in several malignancies, including B-cell acute lymphoblastic leukemia and lung cancer [4]. Unfortunately, to date, no immunotherapeutic approach has shown a survival benefit in glioblastoma patients over chemoradiotherapy [5,6,7,8]. The blood–brain barrier (BBB) and the heterogeneity of glioblastomas are major clinical obstacles to the success of these therapeutic approaches [9].

In this review, we will examine the ongoing challenges of glioblastoma treatment and identify immunotherapeutic approaches that could treat glioblastoma, including Isocitrate dehydrogenase (IDH), Epidermal Growth Factor Receptor (EGFR), and Telomerase reverse transcriptase (TERT) peptide vaccines.

## 2. The Ongoing Challenges of Glioblastoma Treatment

The treatment of glioblastomas with immunotherapy is fraught with challenges due to the location and nature of these tumors [10]. 

### 2.1. Blood–Brain Barrier

Only small lipophilic molecules can passively diffuse through the BBB, while others require transport mechanisms to be able to cross this barrier [11]. This latter regulates the access and activation of immune cells into the central nervous system (CNS) to limit potential neuroinflammation. The BBB remains an obstacle to the administration of systemic immunotherapies in glioblastoma after surgery and standard therapy; it also limits T-cell access to the tumor. To overcome these challenges, chimeric antigen receptor (CAR T) cells are administered by intracranial infusion [12]. 

The widespread observation of radiographic contrast material, typically unable to penetrate the healthy brain, accumulating in nearly all GBM cases, has led to the prevailing belief that the BBB is universally disrupted in GBM patients. Consequently, some argue that considering drug distribution across the BBB is unnecessary when designing treatments for GBM. Nevertheless, contrary evidence from clinical studies highlights the significant presence of tumor burden with an intact BBB in GBM patients. It is increasingly evident that drugs with limited BBB permeability fail to effectively target this portion of tumor cells [13]. Currently, trials are underway to locally compromise the BBB with attempts to disrupt it ultrasonically via a device implanted in the tumor (e.g., NCT04528680) [14]. Nevertheless, Alexander H. Lee et al. demonstrated that this barrier does not prevent the activity of programmed cell death protein 1 (PD-1) antibodies blocking at the tumor site. After treatment of tumor tissue, they detected modifications in T-cells and dendritic cells (DC) after neoadjuvant PD-1 blockade [15]. 

In mice, the combination of LIPU and anti-PD-1 therapy resulted in a longer median overall survival (58 days versus 39 days with anti-PD-1 therapy alone), although the difference was not statistically significant [16]. Moreover, in mice treated with both LIPU and CAR T-cell therapy, there was enhanced delivery of CAR T-cells in the central nervous system (CNS) and a longer median overall survival (mOS) compared to those treated solely with CAR T-cell therapy [16].

### 2.2. Immunotherapy Targeting the Tumor Microenvironment

Glioblastomas are highly infiltrative tumors characterized by intra-patient histological and molecular heterogeneity, making them difficult to classify. Intrinsic intra-tumor heterogeneity is mostly assigned to the complexity of the glioblastoma tumor microenvironment (TME) and its immune evasion capabilities [17]. Glioblastomas with an immunosuppressive microenvironment are often called “cold tumors”. These tumors display low T-cell infiltration due to the BBB, resulting in resistance to therapies including immune checkpoint inhibitors [18]. In addition, these high-grade gliomas are characterized by the absence of tumor antigens, low antigen presentation, and a high accumulation of immunosuppressive cells [19,20]. This facilitates immune evasion [21,22,23]. To enhance antitumor immunity and improve glioblastoma therapies, it is possible to transform cold tumors into warm tumors that are more immunogenic through combinatorial approaches [20].

**Figure 1:**
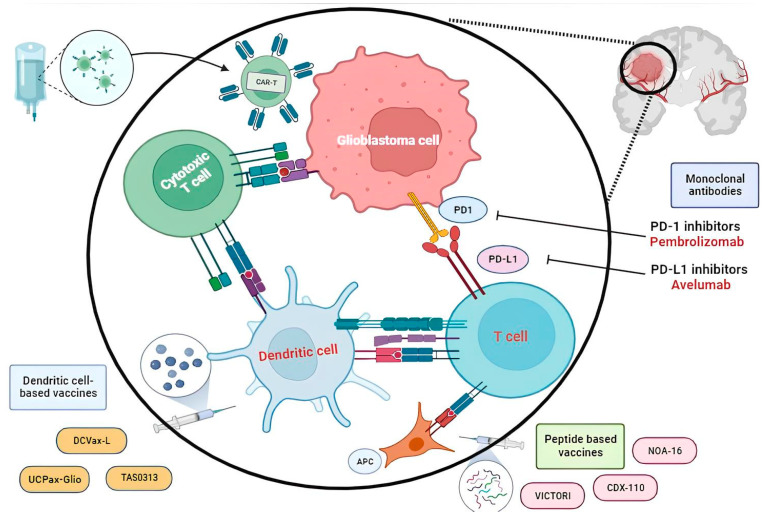
Current immunotherapeutic approaches for the treatment of glioblastoma.

Advances in knowledge of the glioblastoma tumor microenvironment have led to the identification of several potential therapeutic targets and the development of numerous immunotherapies, such as peptide or dendritic vaccines, chimeric antigen receptor (CAR)—modified T-cells, and checkpoint inhibitors (Figure 1). 

The efficacy of immunotherapies in glioblastoma depends on their ability to cross the BBB without major adverse effects. In addition, to overcome the advanced immunosuppressive tumor microenvironment and the heterogeneity of glioblastoma. 

Among the various immunotherapeutic approaches studied to target glioblastoma, we focus specifically on vaccines in this review.

## 3. Vaccine Therapy

Several vaccines targeting glioblastoma are currently under development. Dendritic cell-based vaccines use autologous cells collected by preparing them in vivo with tumor antigens from the patient. They are administered intradermally. Whereas peptide-based vaccines are tumor-specific antigens transported to patients for presentation to T-cells and stimulation of an immune response [24]. Clinical trial numbers were accessed from https://clinicaltrials.gov/ (accessed on 21 April 2024) (Table 1).

### 3.1. Peptide Vaccines

Peptide vaccines are generally about 8–30 amino acids and include a specific epitope of a tumor-associated antigen that elicits immune responses, resulting in an effective anti-tumor T-cell reaction [25]. Given the heterogeneity that characterizes tumor antigens, immunoresistance to a vaccine targeting a single antigen is frequently encountered. Trials are underway to develop vaccines targeting multiple antigens [20].

#### 3.1.1. Wilms Tumor 1

In glioblastoma, Wilms tumor 1 (WT1) is recognized as a tumor-associated antigen (TAA). Clinical trials have demonstrated the safety and tolerability of the WT1 peptide-based vaccine in humans, as well as the induction of WT1-specific IgG antibodies [26,27,28]. In this context, research is still ongoing [29,30].

#### 3.1.2. Survivin

Survivin is an apoptosis-inhibiting protein [31]. A phase 1 trial has shown that the vaccine based on survivin mimetic peptide (SurVaxM) is safe and well tolerated. A Phase 2 trial is currently ongoing to assess the efficacy of this vaccine in newly diagnosed glioblastoma (NCT02455557) [32]. OS and PFS were improved compared to historical controls [33,34]. To manage patients with rGBM, SurVaxM is being studied in combination with pembrolizumab (NCT04013672) [35,36].

#### 3.1.3. Cytomegalovirus

A study of glioblastomas revealed immune reactivity against CMV in all patients [37,38,39,40]. This finding prompted researchers to develop a peptide vaccine against this virus. The vaccine targets CMV’s major structural protein (pp6537). Two therapeutic approaches have been tried: the use of pp6537-stimulated dendritic cells (DCs) [41] and a CMV-specific CD8+ T-cell expansion strategy [42,43]. 

The trials were safe, with encouraging results. The first trial using pp65 DC CMV vaccines showed an mOS of 41.1 months vs. 19.2 months [43,44] (NCT00639639), while the second demonstrated that vaccination of newly diagnosed GBM patients with CMV pp65 RNA-loaded DCs in combination with GM-CSF as an adjuvant, followed by chemotherapy (TMZ), led to an improvement in survival compared to the placebo group that received TMZ alone (mOS = 34 months and mPFS = 31.4 months) (NCT02465268) [39,45].

In the third trial (ELEVATE), after pretreatment with tetanus and diphtheria toxoid, forty-three patients received CMV pp65 DC vaccine (NCT02366728). OS was 3 years in 34% of cases [44,46].

#### 3.1.4. Neoantigen

Neoantigen expression is absent in normal tissue. Neoantigens are T-cell epitopes derived exclusively from tumor specific somatic DNA mutations. This gives them very high immunogenicity [47]. Recently, studies have been carried out on vaccines against neoantigens in GBM patients. The first phase of a clinical trial showed that vaccination of newly diagnosed glioblastomas with a personalized neoantigen vaccine (NeoVax) induced neoantigen-specific circulating CD4+ and CD8+ T-cell responses and neoantigen specific intratumoral T-cell infiltration (NCT02287428) [48,49]. However, an immune response was not observed in patients who received concomitant dexamethasone-based steroid therapy [48].

Mixed autologous and allogeneic vaccines represent a promising approach for GBM treatment. These vaccines use both the patient’s own immune system and donor-derived immune cells to initiate a strong anti-tumor response. The autologous component involves using the patient’s own tumor cells to train the immune system to specifically recognize and attack cancer cells.

On the other hand, the allogeneic element incorporates immune cells from a donor source, enhancing the immune response with a wide range of antigens potentially expanding the range of targeted tumor cells. This mixed approach capitalizes on the strengths of both autologous and allogeneic vaccination techniques, with the aim of overcoming the limitations of each method when used alone. The typical example of an autologous/allogeneic vaccine used in GBM is GLIOVAC/ERC1671. In their study, Schijins and his collaborators reported a significant increase in overall survival in the GLIOVAC regimen compared to the control group [50].

#### 3.1.5. Multipeptide Vaccines

Given the ability to escape antigen when targeting a single peptide, there has been a focus on developing vaccines that target multiple tumor antigens concurrently. Promising results have been achieved in a phase 1/2 study assessing the effectiveness and safety of TAS0313, a cancer peptide vaccine targeting eight TAAs (antigens derived from EGFR, LCK, KUA, PTHRP, MRP3, WHSC2, SART2, and SART3) [51].

In the second phase of another trial, administration of the ICT-107 vaccine, composed of six TAAs (IL13Rα2, MAGE-1, AIM-2, HER-2, TRP-2, gp100) pulsed into dendritic cells resulted in a significant increase in progression-free survival in GBM patients [52]. This trial was randomized, double-blind, and controlled (NCT01280552).

Currently, in the context of the treatment of progressive GBM, a third vaccine (EO2401) is being explored. It targets Oncomimics: three bacterial peptides mimicking tumor antigens [53]. Trials are in phase 1b/2a (NCT04116658).

### 3.2. Dendritic Cell-Based Vaccines

In the immune system, dendritic cells (DCs) are the most potent antigen-presenting cells (APCs). They play an important role in the T lymphocyte response [54]. After activation of CD8+ T-cells via major histocompatibility complex (MHC) class II proteins [55], CD8+ T-cells are able to identify and eradicate glioblastoma cells presenting specific antigens on their surface [56].

DCVax is the most crucial cell-based vaccine. In this approach, dendritic cells (DCs) generated ex vivo from the patient’s peripheral blood are pulsed with different sources of tumor-associated antigens, including antigen peptides and autologous tumor lysates. Indeed, DCVax has enabled personalized targeting of several tumor antigens to achieve a potentially durable response.

A Phase III clinical trial evaluated the safety and efficacy of DCVax^®^-L, a vaccine based on autologous dendritic cells pulsed with tumor lysate antigen, in the treatment of newly diagnosed glioblastoma multiforme (NCT00045968). Adverse events were mild, and median overall survival was 23.1 months compared to 13–15 months in patients receiving standard therapy alone [57,58]. Numerous obstacles specific to dendritic cells (DCs) have hindered the efficacy of DC-based vaccines, including the suppressive immune environment. This can prevent dendritic cells from enhancing interferon-γ production and natural killer (NK) lymphocyte cytotoxicity. To overcome this challenge, the use of vaccine adjuvants is desirable and is being incorporated to enhance NKT and DC activity in the treatment of GBM [59,60,61]. 

Recently, researchers have developed heat shock protein (HSP)-based anti-cancer vaccines that utilize HSPs to present tumor specific antigens, stimulating a potent anti-tumor-immune response [62]. Those HSP-based immunotherapies have the ability to stimulate tumor antigens uptake by antigen-presenting cells, including DCs, to allow T lymphocyte-mediated cytotoxic death [62]. The HSPC-96 is one of the most widely explored HSP vaccines. HSPC-96 vaccine comprises patient-specific tumor antigens linked to the HSP gp-96 protein. In a second phase clinical trial, forty-one patients with recurrent GBM were administered the HSPPC-96 vaccine (NCT00293423). Results showed a mean survival of 42.6 weeks vs. 19.1 weeks in control patients [63]. Another clinical trial evaluated HSPPC-96 and bevacizumab, but no improvement in overall survival was noted (Phase 2) [64]. 

While there is substantial evidence supporting the feasibility and safety of integrating DC vaccines into standard therapy for glioblastoma patients, along with potential extensions in overall survival, the production of these vaccines requires considerable time and financial resources. Additionally, despite the demonstrated biological activity of DC vaccines, the clinical advantages are not consistently significant enough. Surely, further clinical trials are imperative to assess the potential survival advantages of DC vaccines, either independently or in combination with other immunotherapies [52,65].

### 3.3. Biomarkers as Potential Targets for Vaccine Therapy

Several proteins are commonly altered in glioblastoma, including IDH1, EGFR, TERT, tumor protein p53 (TP53), phosphatase and tensin homolog (PTEN), neurofibromatosis type 1 (NF1), platelet-derived growth factor receptor α (PDGFRA) and retinoblastoma 1 (RB1) [25].

#### 3.3.1. IDH

Isocitrate dehydrogenase (IDH) plays a crucial role in several mechanisms included in the Krebs cycle by stimulating the oxidative decarboxylation of isocitrate to alpha-ketoglutarate. In multiple human tissues, including brain tissue, IDH is implicated in the production of NADPH, which accumulates at the cytosolic level. At the molecular level, IDH1 resides at 2q33, and IDH2 is located at 15q26. These genes play essential roles in shielding cells from replicative senescence by decreasing lipid peroxidation and oxidative DNA damage [66,67]. The IDH1 and IDH2 proteins exhibit a high degree of similarity between them, with approximately 70% similarity in human cells. They are encoded by separate genes, *IDH1* located on chromosome 2q33 and *IDH2* on chromosome 15q26 [68,69]. 

Alterations at this metabolic level result in a reduction and disruption of *IDH* function. Mutations that impact isocitrate dehydrogenase are characterized as hotspot mutations and have assumed a crucial role in guiding the diagnosis, prognosis, and prediction of gliomas since their initial discovery. These are codon 132 for *IDH*1 (*IDH*1^R132^ in 90% of cases) and codon 172 for *IDH*2 (*IDH*2^R172^ in 10% of cases), which affect the genes encoding for IDH1 (cytoplasmic form) or IDH2 (mitochondrial form). These mutations result in the substitution of *Arg*^132^ → *His* at the protein’s active site, leading to profound changes in the epigenetic profile. This, in turn, causes a decrease in the production of NADPH, which protects the cell against oxidative stress, resulting in an increased production of D-2-hydroxyglutarate, promoting angiogenesis [70,71]. They play an essential role in classification and prognosis stratification.

Mutation in *IDH* occurs during the initial stages of glioma development [27]. They are also present in recurrent gliomas [72,73]. Glioblastomas that have the normal, non-mutated *IDH* gene are mainly primary or de novo tumors; which typically emerge in individuals aged over 50 years. These patients exhibit a brief clinical history, often less than three months before diagnosis, and they do not have any pre-existing lower-grade gliomas. Considering the significance of the *IDH* mutation, targeting mutant *IDH1/2* shows great promise as a treatment approach for gliomas [74,75,76]. Numerous clinical trials are currently in progress to explore this avenue.

According to several studies, genes responsible for the production of chemokines that recruit immune cells are suppressed in *IDH* mutant gliomas [77,78,79,80].

In 2014, Schumacher et al. were the first to try to develop vaccines against mutant IDH. A peptide composed of twenty amino acids was inoculated into mice, covering part of the mutated catalytic pocket of the IDH enzyme. The results showed a strong immune response from helper T-cells specific to this mutation [74].

In humans, NOA-16 was the first phase 1 trial of the IDH1 Peptide Vaccine (NCT02454634). Newly diagnosed patients with grade 3 and 4 IDH1R132H astrocytomas were enrolled [81,82]. The IDH vaccine induced an immune response represented by pseudoprogression. But it turned out that this could be a late response to radiotherapy before the vaccine [81]. The aim of this trial was to evaluate the safety and tolerability of the IDH1 vaccine (NCT02454634). The majority of patients presented mild adverse effects. The phase 2 trial will attempt to evaluate the vaccine’s efficacy.

In order to overcome immune evasion and achieve promising results, a combinatorial approach of mutant IDH inhibitors with vaccine therapy or immune checkpoint inhibitors is required [77,78,79,80]. 

#### 3.3.2. EGFR

EGFR, a pivotal tyrosine kinase protein within cellular signaling networks, becomes activated upon binding with its ligand EGF, instigating critical pathways involved in cell proliferation that sustain cell survival by regulating the cell cycle progression. These pathways include the MAPK pathway, which features proteins like RAS, RAF, and MEK, as well as factors in the PI3K-AKT-mTOR pathway that enhance cell survival by inhibiting apoptosis. Activation of these signaling routes can compromise the integrity of G1-S cell cycle checkpoints, ultimately leading to excessive proliferation [83,84].

The predominant genetic alterations cited in glioblastomas are related to alternative splicing, rearrangements, and amplification of the *EGFR* gene [85]. Genetic rearrangement and amplification are the most common traits expressed in GBM (40–50%). The tyrosine kinase protein (epidermal growth factor receptor) is encoded by a gene called *EGFR* located at 7p11.2. Mutations caused by histone modifications occurring at this level are detected, especially in primary GBM. The formation of EGFRvIII due to truncated exons 2 and 7 leads to the absence of the extracellular ligand-binding site [86,87]. The presence of mutation and amplification of *EGFR* has been classified as prognostic indicators due to their prevalence in GBM [88,89].

*EGFR* amplification in isolation does not exhibit a prognostic influence on survival, as evidenced by prior studies. However, some studies reported a notable reduction in survival duration among patients manifesting EGFR overexpression. Furthermore, the prognosis becomes significantly poor when this genetic alteration is coupled with amplification [90,91,92]. Patients with mutated *EGFR* have a low life expectancy (10 months) compared to patients without the mutation, whose life expectancy is 1.4 years. Therefore, the presence of this mutation leads to a poor prognosis for patients with glioblastoma [86,87].

EGFRvIII is a ligand-independent and constitutively active splice variant of *EGFR*, promoting tumor growth and resistance to chemotherapy [93,94]. Given the high expression rate and oncogenic characteristics of this biomarker, it could be an ideal target for glioblastoma immunotherapy.

Rindopepimut (CDX-110) is a peptide vaccine delivered via injection, aiming at EGFRvIII, comprising a 14-unit peptide spanning EGFRvIII’s extent [95], linked to the non-specific carrier protein KLH (control). CDX-110 was designed with the aim of stimulating the production of polyclonal antibodies specific to EGFRvIII. These antibodies have shown the ability to provoke potent anti-tumor responses against EGFRvIII-positive cells after gross total resection and chemoradiation [96]. 

Studies on an animal model vaccinated with rindopepimut have shown an enhancement in the EGFRvIII-specific humoral response, which may inhibit tumor growth and improve overall survival [97,98].

In the VICTORI trial (phase I), newly diagnosed cases of glioblastoma were vaccinated with rindopepimut-pulsed, monocyte-derived dendritic cells. EGFRvIII-mediated immune activation was observed in most patients, but unfortunately, there was no statistically significant improvement in survival [99].

The efficacy of rindopepimut in EGFRvIII-expressing GBM is currently being tested in three trials (Phase 2): ACTIVATe I, ACTIVATe II and ACTIVATe III; vaccination after surgery; and treatment with TMZ radiochemotherapy (NCT00643097).

In all these trials, vaccination was accompanied by the use of colony-stimulating agents like granulocyte-macrophage colony-stimulating factor (GM-CSF). These agents enhance the population of white blood cells and platelets in the bone marrow or peripheral blood [94,96,100]. The trials have confirmed the safety of this approach and have shown a notable increase in survival rates among vaccinated patients [94,96,100]. 

In the phase III ACTIVATe IV trial (NCT01480479), the addition of the rindopepimut vaccine to chemotherapy did not improve patient survival [101]. Overall survival was 20.1 months, compared to 20.1 months using the control (Keyhole Limpet Hemocyanin). 

However, the combination of rindopepimut with bevacizumab in the phase II ReACT trial (NCT01498328) reported a slight survival advantage in patients with recurrent glioblastoma (12 months vs. 8.8 months in the control arm) [7,102]. Indeed, in these trials, a loss of antigens was observed in post-treatment samples, indicating immunosuppression of glioblastoma following effective treatment [103,104].

To date, the rindopepimut vaccine has not been validated; however, CAR-T studies targeting EGFRvIII are underway [105].

#### 3.3.3. TERT

TERT protein is a ribonucleoprotein enzyme complex found in eukaryotic organisms that operates on a native RNA molecule as a template to preserve and elongate telomeres. Consequently, it can increase the potential for cell divisions and function as an RNA-dependent DNA polymerase; it catalyzes the 3′ extension of chromosomes by adding hexamers; that counteract the depletion of these DNA segments by generating duplicate telomeric sequences in actively dividing cells [106].

Mutations occurring in *TERT*, not only within its coding sequences but also within its regulatory regions, could potentially contribute to the development of cancer. Furthermore, mutations within the *TERT* promoter region can reactivate the telomerase enzyme, thus preventing the shortening of telomeres and ultimately resulting in the immortalization of cancerous cells, As a result, evading replicative senescence [107,108]. It is a viability indicator because, in each cell division, the size of telomeres decreases. In glioblastomas, this mutation is observed in 80% of cases [109,110]. 

The majority of glioblastoma cases (70–90%) exhibit mutations in the *TERT* promoter, specifically the C228T or C250T variants, which are located at positions −124 bp and −146 bp before the *TERT* translation start site (5p15.33).These mutations involve the conversion of cytosine to thymine at two specific positions: 1295228 C > T and 1295250 C > T, as documented by Arita et al. in 2016 [111].

Numerous studies have shown that the effect of *TERT* mutations on prognosis depends on an assortment of variables, including age, tumor histology, the absence of *IDH* mutations, and the existence of an unmethylated O^6^-methylguanine-DNA-methyltransferase (*MGMT*) promoter [111,112,113]. 

The presence of a *TERT* promoter mutation is specifically associated with a more favorable prognosis in glioblastomas with *IDH* mutations. However, patients who have both unmethylated MGMT promoters and *TERT* promoter mutations tend to have the poorest prognosis [113]. Additionally, the prognosis appears to worsen with the presence of *TERT* promoter mutations in glioblastomas with an *IDH* wild-type status [109].

Given the prognostic value of the *TERT* promoter mutation, several researchers have attempted to develop therapies targeting TERT activity, such as small-molecule inhibitors and vaccines, which are currently being explored [114]. 

In a phase I/II trial, Vik-Mo and colleagues successfully transfected dendritic cells with RNA extracted from autologous cancer stem cell cultures, along with mRNA and hTERT. These transfected cells were administered following the completion of postoperative chemo-radiotherapy. All seven participants in the trial generated an immune response without exhibiting signs of autoimmunity or significant toxicity (NCT00846456). They also showed significantly longer progression-free survival than unvaccinated patients (694 days vs. 236 days), and five patients were still alive after a two-year follow-up [115].

Nucleic acid-based vaccine development involves creating plasmids that encode TSA and cytokines. This process promotes recognition and activation of the CD8+ T-cell response [102]. The aim of an active Phase 1/2 clinical trial was to investigate the safety, immunogenicity, and initial efficacy of INO-5401 and INO-9012. Both of them are DNA-based vaccines administered alongside cemiplimab (REGN2810), radiation, and chemotherapy with temozolomide to patients with newly diagnosed glioblastoma (NCT03491683). INO-5401 comprises three DNA plasmids targeting human TERT, WT1 antigen, and prostate-specific membrane antigen (PSMA). INO-9012 is a DNA plasmid engineered for the expression of human interleukin-12 (IL-12). Given their ability to penetrate the nucleus and present antigen to MHC, these vaccines are the most effective nucleic acid-based vaccines.

UCPVax-Glio is a vaccine based on telomerase-derived helper peptides designed to elicit strong Th1 CD4 T-cell responses. Phase II trial of this vaccine is still ongoing (NCT04280848) in glioblastoma patients [116]. Administration of this vaccine will begin one month after the end of concomitant radiochemotherapy. 

B7-H4 mediates immune evasion in the tumor microenvironment. Its activation blocks immune responses [117]. In addition, it is highly expressed in glioblastomas [118]. In 2018, a phase 2 clinical trial [119] evaluated a pulsed cell vaccine (DCV) with glioblastoma stem cell antigens. Patients were stratified according to *IDH* and *TERT*p mutation status and also B7-H4 expression. After vaccination, overall survival improved in the *IDH1*-wt/*TERT*p-mut glioblastoma group, which had the lowest B7-H4 expression among the other two groups. This finding makes *TERT*p-mutated glioblastoma patients preferential candidates for DCV treatment. 

## 4. Clinical Side and Limitations

Several factors can limit the efficacy of immunotherapy, including the low percentage of glioblastoma patients enrolled in clinical trials (Only 11%) [120]. This is due to a lack of knowledge about trial availability and the fact that patients’ physical or cognitive symptoms may reduce their ability or willingness to travel [121]. Indeed, a study involving 57 patients showed that travel time of less than one hour was significantly associated with a greater willingness to consider participation in a clinical trial [122]. On the other hand, efforts are underway to enhance clinical trial participation by optimizing strict eligibility criteria [123]. To enhance immunotherapy for glioblastoma patients, many other limitations must be considered, notably the slowness and inefficiency of clinical trials [124]. 

Glioblastoma cases are eliminated from phase 1 of clinical trials assessing new agents without solid justification [124]. Given that these patients are typically in good health, their greater inclusion in phase I oncology trials could expedite the discovery of promising new agents, enabling earlier-stage evaluation [125]. To identify promising agents and develop them, it is essential to set up more efficient clinical trial networks focused on these studies.

Most glioblastoma treatments have been evaluated in uncontrolled single-arm studies using PFS or OS as primary endpoints, often compared with contemporary or historical controls. However, limitations such as inadequate historical controls and the absence of biomarkers to enrich patient populations or predict treatment outcomes have led to numerous unsuccessful phase II to III transitions for various treatments [126]. Additionally, the lack of understanding behind these failures hinders the ability to learn and improve trial designs in the future. 

On the clinical side, moving from single-center, single-arm studies to randomized, controlled, and sufficiently powered clinical trials can make a significant contribution to the development of more robust therapies by improving reproducibility of results and without wasting valuable financial resources [127].

## 5. Potential Solutions to Overcome the Immunotherapy Challenges of Glioblastoma

Following the success of CAR-T cell therapy in haematological tumors, studies have been carried out in glioblastoma [128]. This approach may yield promising results compared with other therapies. The use of immune cell trafficking enables better penetration of the BBB [129]. Moreover, this therapy does not depend on the immune response, which is suppressed in glioblastoma. Tumor cells are killed directly once bound to the receptor [130,131,132].

The immunosuppressive microenvironment, intratumoral heterogeneity, antigen loss, and limitations of mouse models of Glioblastoma also represent challenges to the success of this therapy. To overcome these obstacles and improve the clinical response and efficacy of glioblastoma therapy, several solutions have been proposed (Table 2).

As mentioned above, efforts have been made to address the challenges facing vaccine therapy for GBM. Nanoparticles (NPs) are being developed to overcome the BBB. Several classes of NPs are being explored, such as lipid-based nanoparticles, polymeric nanoparticles, inorganic nanoparticles, and biological nanoparticles [191]. One approach involves modifying NPs with ligands or substrates that bind to receptors or transporters highly expressed on CNS endothelial cells, thereby inducing transcytosis. Examples include transferrin and glucose transporter 1 (GLUT1), which are highly expressed on both CNS endothelial cells and tumors [192,193,194,195]. 

Another strategy involves utilizing cells with innate tumor-tropic homing ability, such as mesenchymal stem cells (MSCs) and certain white blood cells, to transport NPs across the BBB [196,197,198,199,200,201]. While this approach has yet to be tested in animals with intact BBB, it shows promise in orthotopic xenograft glioma mouse models. Additionally, combination approaches are devised to disrupt or bypass the BBB using methods like intranasal delivery, convection-enhanced delivery, and focused ultrasound [196]. These methods, combined with NPs designed for enhanced distribution and prolonged release, aim to increase therapeutic retention at the disease site. Furthermore, modifying NPs with polyethylene glycol (PEG) can increase circulation time by evading the reticuloendothelial system (RES) clearance, although its impact on BBB penetration varies [196,202,203].

Other strategies, such as incorporating antiphagocytosis signals like CD47, show promise in overcoming rapid RES clearance [196,202,203].Complex NPs combining multiple strategies and exhibiting additional effects like magnetothermal or photothermal properties are also under evaluation. Overall, continued research and development of NPs as therapeutic delivery vehicles for CNS tumors hold significant promise for clinical translation [196].

The tumor expresses various chemokine ligands such as chemokine (C-C) ligand 17 (CCL17) and chemokine (C–C) ligand 22 (CCL22), which facilitate recruitment of CC Chemokine Receptor 4 (CCR4+) regulatory T-cells (Tregs), while C-X-C motif chemokine receptor 3 (CXCR3)-with its ligands, C-X-C motif chemokine ligand 9 (CXCL9) and C-X-C motif chemokine ligand 10 (CXCL10)—promotes trafficking of cytotoxic lymphocytes into the GBM tumor site. To enhance the localization of T-cells to tumors, CAR-T cells can be engineered to express corresponding chemokine receptors [134,138,139]. For instance, CD70-specific CAR-T cells expressing CXCR1 and CXCR2 exhibited improved migration and antitumor efficacy in murine models of GBM [140].

In order to avoid recruitment difficulties in the bloodstream, another approach involves directly infusing CAR IL-13Rα2 into the tumor by intralesional or intrathecal infusion. These assays showed tumor regression and persistence of CAR-T cells at sites of intralesional perfusion and tumor progression. Intravenously infused CAR-T cells were able to circulate, penetrate the BBB and reach the targeted tumor tissue [142,204].

Consequently, the optimal route for CAR-T cell delivery requires further investigation, with current exploration focusing on intravenous, intratumoral, and intraventricular routes [169]. When CAR-T cells migrate to the tumor site, they are faced with a highly immunosuppressive microenvironment. IL-6 and TGF-ß are the main TME molecules known as contributors to immunosuppression, present potential targets for monoclonal antibody therapies that could be administered concurrently with CAR-T therapy [147]. 

Moreover, CAR-T cells could be modified to produce antibodies, cytokines, or adjuvants [159]. This includes the secretion of cytokines like interleukin (IL): IL-12, IL-15, IL-18, and IL-21 by T-cells redirected for universal cytokine-mediated killing (TRUCKs), can enhance their proliferative activity and reshape the TME while also attracting nearby anti-tumor immune cells [159,161].

For instance, in a preclinical glioma model, IL-15 expression led to increased persistence and proliferative capacity of IL-13Rα2-CAR-T cells, resulting in enhanced anti-tumor activity [160]. CAR-T cells can be engineered to produce antibody-like proteins, potentially enhancing their ability to recognize tumor-associated antigens and strengthening immune responses such as antibody-dependent cell-mediated cytotoxicity [149]. Moreover, various strategies have been employed to enhance CAR-T cell survival in cancer immunotherapy, including targeting checkpoints. The initial approach utilizes monoclonal antibodies against PD-1/PD-L1 to block co-inhibitory signals, thereby restoring cytokine production and boosting the survival of CAR-T cells [156,164]. The second is employing gene-editing technologies to target co-inhibitory molecules, and the last strategy involves expressing PD-1 switch receptors [162,163].

One study compared various checkpoint blockade approaches in IL-13Rα2- and EGFRvIII CAR-T cells. It found that reversing anergy in CAR-T cells, when used to treat murine and canine gliomas, led to significant reductions in tumor growth. Specifically, PD-1 blockade benefited EGFRvIII CAR-T cells, while CTLA-4 blockade enhanced the efficacy of IL-13Rα2 CAR-T cells [165]. Shen et al. discussed the concurrent administration of checkpoint inhibitors with CAR-T cells in GBM [166]. They demonstrated that CAR-T cells engineered to secrete PD-1-blocking single-chain variable fragments (scFV) locally showed superior performance compared to CAR-T cells combined with systemic PD-1 blockade using monoclonal antibodies [167]. This highlights the importance of localized PD-1 blockade. Moreover, CAR constructs have been engineered by deleting PD-1 through CRISPR Cas9 technology, resulting in CAR-T cells that are less susceptible to exhaustion, thus improving their effectiveness against glioblastoma in murine models [168,169,170,171,172]. Additionally, the inhibitory signaling domain of PD-1 has been replaced with a stimulating domain, such as that derived from CD28, creating PD-1 switch receptors. These receptors receive activating co-stimulatory signals when engaged by PD-L1, potentially enhancing CAR-T cell function [138,156,169,205]. However, a drawback to this approach is the potential for heightened toxicity due to uncontrolled CAR-T cell activation without feedback and attenuation mechanisms [159,168].

Similar strategies target other T-cell inhibitory receptors such as CTLA-4, T-cell immunoglobulin and mucin domain 3 (TIM-3) and lymphocyte activation gene 3 (LAG-3), which are under investigation [139]. Downregulation of PD-1, TIM-3, and LAG-3 resulted in quick and continuous activation of CAR-T cells but a reduced memory cell pool, potentially leading to a shorter treatment time [139,141]. Further research is needed to assess the advantages of immune checkpoint blockade [139,141]. 

To conquer the local suppression of the immune system, research has also indicated that specific agonists targeting TAM, such as poly-ICLC, resquimod, and imiquimod, can serve as vaccine enhancers to boost the effectiveness of vaccine treatments. This approach has shown promise in extending the mPFS of GBM patients to 21 months following diagnosis [173]. The mechanism underlying this effect involves the ability of these agents to repolarize TAM [174]. Personalized vaccination therapy targeting neoantigens is one approach used to make the tumor microenvironment hotter [175]. In addition, several data points suggest that the gut microbiota plays a role in regulating immunity and metabolism within the GBM microenvironment, providing a potential route for therapeutic intervention to modulate the immunosuppressive MET of GBM [176].

As previously mentioned, the presence of both intra- and inter-tumoral heterogeneity and antigen loss pose significant challenges in CAR-T cell therapy for GBM [179]. Efforts are underway to overcome these challenges, with strategies focusing on engineering CAR-T cells able to target multiple tumor-associated antigens (TAAs) or adapt to varying antigens. This encompasses the development of bi-specific or even trivalent CARs, as well as tandem CARs capable of targeting multiple antigens simultaneously, as evidenced in preclinical trials [181,182].

Another promising approach involves utilizing Smart CAR-T cells, which are engineered to be multi-targeted and programmable. These encompass synthetic Notch (SynNotch) CARs, universal CARs, and split universal programmable (SUPRA) CARs. This new-generation of CARs incorporates adaptable receptors capable of concurrently targeting various tumor antigens with both high efficacy and precision, while maintaining low toxicity levels [183]. Universal CARs are activated through the binding of the extracellular adaptor domain to a soluble antigen-targeting ligand, such as monoclonal antibodies (mABs), attached to tumor cell surfaces. This facilitates selective and adaptable targeting of multiple tumor antigens based on the chosen soluble ligand [183]. Conversely, SUPRA CARs utilize a single-chain variable fragment (scFv) adaptor molecule and a universal receptor for flexible targeting of multiple antigens without the need for complex reengineering. This option enables the adjustment of tumor treatment based on changes in antigen expression within a patient’s tumor [183,184].

Polytherapeutic approaches offer additional potential by targeting treatment-resistant tumorigenic populations and addressing challenges associated with tumor heterogeneity [187]. Combinations with other immunotherapeutic modalities or classical cancer therapies like radiotherapy or chemotherapy are also feasible. Radiotherapy for gliomas has been shown to synergize with immunotherapy. Following this approach, there is a notable upregulation of neoantigens on tumor cell surfaces, increasing targets for T-cells [148,189]. For example, natural killer cell receptor group 2 member D (NKG2D) CAR-T cells, when combined with intracranial radiotherapy, significantly reduced tumor burden and improved survival, resulting in upregulating NKG2DL following radiotherapy [188]. Additionally, this treatment induces the release of damage-associated molecular patterns (DAMPs) at the tumor site, which, in turn, enhances CAR-T cell infiltration by increasing chemokine expression [148,189]. Moreover, chemotherapeutic agents like cyclophosphamide and doxorubicin can also augment T-cell infiltration into solid tumors and improve antigen presentation [190].

Regarding glioma stem-like cells (GSCs), we assume that they contributed to treatment failure in GBM, primarily due to their higher resistance to drugs and radiation, in addition to their capacity to promote tumor recurrence [129]. Targeting GSCs in glioma-bearing mice has demonstrated efficacy in inhibiting glioblastoma formation and prolonging survival. CD133 has been identified as a potential target antigen due to its association with the GSC phenotype [185,186].

Moreover, dysregulation of glioma stem cell pathways by targeting miRNA could provide an effective and personalized strategy against TMZ-resistant glioblastoma in the future [206,207]. Given that GSCs play a significant role in disease recurrence and metastasis, their elimination is crucial for developing curative treatments [208]. Future strategies should focus on combining anti-GSC therapies with other therapeutic approaches [8].

## 6. Conclusions and Future Directions

Despite advances in the management of glioblastoma, this tumor remains the most common and deadly malignant glioma in adults, with a median survival of 15 months. As previously mentioned, immunotherapeutic approaches based on vaccines, chimeric antigen-receptor T-cells, and checkpoint inhibitors have demonstrated limited effectiveness in the treatment of GBM. In this review, we highlight several hurdles facing immunotherapy, including tumor heterogeneity, the immunosuppressive environment, and blood–brain barrier. To address this issue, we have listed the main proposed solutions, such as nanoparticle technology, CAR-T cell therapy, and polytherapeutic approaches. The emergence of nanoparticles provides a new direction for the efficient, targeted delivery of drugs to overcome BBB. CAR-T cells are also able to circulate and reach the targeted tumor tissue. Moreover, targeting checkpoints and interleukins have been employed to enhance CAR-T cell survival in cancer immunotherapy.

In the future, it is essential to deepen our understanding of the molecular pathology of these tumors, signaling pathways inducing gliomagenesis as therapeutic targets, the mechanisms behind immunosuppression in GBM, and to improve the potency and efficiency of tumor-specific antigenic profiles. On the other hand, a multidisciplinary and collaborative approach involving basic research, translational research, and clinical trials will be essential to transform glioblastoma vaccines into an effective therapeutic reality for patients.

While some vaccines have shown efficacy and safety only in phase I and II trials, the overall results of phase III clinical trials remain disappointing, with no significant improvement in GBM prognosis. Hence, as highlighted by Dr. Patrick Y. Wen, there’s a clear need for more phase III trials with a more efficient design to address this issue [209]. Combining several therapies could be a future direction of tumor vaccine development and is proving highly effective in terms of patient survival [209]. In this review, we identified the main biomarkers known as potential immunotherapeutic targets, and the management of glioblastoma must be personalized and tailored to patients with distinct molecular characteristics to optimize therapeutic outcomes. Moreover, we suggest an open dialogue between researchers, clinicians, and patients to ensure that scientific advancements translate into tangible improvements in the clinical management of glioblastoma.

## Figures and Tables

**Table 1:** Active or completed clinical trials for vaccines in glioblastoma (https://clinicaltrials.gov/ (accessed on 21 April 2024)).

Vaccine	Trial Title	Phase	N	Experience Details	Endpoints	Clinical Trial
Wilms tumor 1 (WT1)	WT1 peptide vaccination for patients with recurrent glioblastoma multiforme (rGBM)	2	21	WT1 peptide	Median progression-free survival (mPFS) = 20 weeks at 6 months (mo) (26-week), PFS rate = 33%	NA (Not available)
	WT2725 in patients with advanced malignancies	1	64	WT1 peptide (WT2725)	Response rate in GBM = 20% OS at 12 mo in GBM = 33%	NCT01621542 Completed
	IMT03 study for Newly Diagnosed (Nd) Malignant Glioma with WT1-W10 Vaccination	1/2	27	WT peptide (W10)	OS = 21.9 mo PFS = 12.7 mo	NA
	INO-5401 and INO-9012 vaccines delivered by Electroporation (EP) and combined with Cemiplimab (REGN2810) in Nd GBM	1/2	52	O6 methylguanine DNA methyltransferase (MGMT) INO-5401 + INO-9012 + Cemiplimab + RT + temozolomide (TMZ)	mOS in MGMT-UN (Unmethylated: 17.9 months vs. 32.5 months in MGMT-M (Methylated)	NCT03491683 Active, not recruiting
Survivin	Evaluation of the Safety and Efficacy of SVN53-67/M57-KLH (keyhole limpet hemocyanin) (SurVaxM) in Survivin Positive Newly Diagnosed Glioblastoma	2	66	SurVaxM + TMZ	PFS at 6 mo in patients treated with at least four doses of peptide vaccine	NCT02455557 Active, not recruiting
	Pembrolizumab Plus SurVaxM for Glioblastoma at First Recurrence	2	40	SurVaxM + Pembrolizumab	PFS at 6 mo	NCT04013672 Active, not recruiting
CMV (cytomegalovirus)	Vaccine Therapy for the Treatment of Nd GBM (ATTAC-I)	1	42	pp65-DC vaccine + tetanus toxoid (Td) preconditioning	Safety and feasability OS = 20.6–47.3 mo vs. 13.8–41.3 mo (*p* = 0.013)	NCT00639639 Completed
	Vaccine Therapy for the Treatment of Newly Diagnosed Glioblastoma (ATTAC- II)	2	175	CMV pp65-mRNA loaded DCs + GMCSF + Td + TMZ	mPFS = 31.4 mo and mOS = 34 mo	NCT02465268 Completed
	Assessment of surmounting limited migration and improving CMV specific DC Vaccines with Adjuvant Tetanus in Nd GBM (ELEVATE)	2	64	pp65 DC vaccine + TMZ vs. pp65 DC vaccine + TMZ + preconditioning	3-year OS = 34% (95% confidence interval (CI) 19–63%) vs. 6% (95% CI 1–42%)	NCT02366728 Completed
Neoantigens	Personalized NeoAntigen Cancer Vaccine With Radiotherapy (RT) Plus Pembrolizumab/MK-3475 Among Newly Diagnosed Glioblastoma Patients	1/1b	56	NeoVax + RT vs. Neovax + RT + Pembrolizumab	Safety and tolerability	NCT02287428 Recruiting
Multipeptide Vaccines	Evaluation of the efficacy of TAS0313 in rGBM	1/2	17	TAS0313	Safety, tolerability and efficacy. mPFS = 2.2 (95% CI, 1.0–2.3) months overall response rate (ORR) = 11.1% (95% CI = 0.3–48.2%)	JapicCTI183824
	Evaluation of the safety and efficacy of ICT-107 in Nd GBM	2b	124	Resection + Chemoradiation + ICT107 vs. placebo	OS = 17 vs. 15 mo hazard ratio (HR) = 0.87; *p* = 0.58) PFS = 11.2 vs. 9.0 mo (HR = 0.57; *p* = 0.011)	NCT01280552 Completed
	First-in-Human, a Multipeptide Therapeutic Vaccine in Patients with Progressive Glioblastoma (ROSALIE)	1b/2a	52	EO2041 + nivolumab vs. EO2041 + nivolumab + bevacizumab	Safety and tolerability	NCT04116658 Active, not recruiting
Dendritic cells	Clinical Trial Evaluating DCVax®-L, Autologous Dendritic Cells Pulsed With Tumor Lysate Antigen to treat newly diagnosed GBM	3	348	TMZ + DCVax-L vs. TMZ + placebo	mOS = 23.1 mo (95% CI 21.2–25.4)	NCT00045968 Active, not recruiting
	Study of a Dendritic Cell Vaccine for Patients with Either Newly Diagnosed or Recurrent GBM	1	39	DC vaccine + GBM stem-like cell lysate	Safety and tolerability mPFS = 8.75 mo in newly diagnosed GBM, 3.23 mo in recurrent GBM mOS = 20.36 mo in newly diagnosed GBM, 11.97 mo in recurrent GBM	NCT02010606 Completed
	Study of DC-Based Therapy Targeting Tumor Stem Cells in patients with GBM and receiving therapy	1/2	20	DC vaccine pulsed with mRNA from glioma stem cells (GSCs)	Safe study, immune response towards the stem-cell like part mOS for treated group: 759 days vs. 585 days for control group progression-free survival 694 days vs. 236 days for unvaccinated patients	NCT00846456 Completed
HSP	Heat Shock Protein Peptide Complex-96 (HSPPC-96) Vaccine for cases with Recurrent or progressive High Grade Glioma	1/2	96	HSPPC-96 vaccine	OS at 6 mo = 90.2% (95% CI 75.9–96.8%) mOS = 42.6 weeks (95% CI = 34.7–50.5)	NCT00293423 Completed
	Randomized Trial assessing the Efficacy of HSPPC-96 Vaccine in the Treatment of rGBM	2	90	HSPPC-96 vaccine+ bevacicumab compared to Bevacizumab alone	OS = 7.5 vs. 10.7 mo (HR = 2.06)	NCT01814813 Completed
IDH	Targeting IDH1R132H in Glioma (WHO Grade III-IV) with IDH1R132H by a Peptide Vaccine Multicenter Trial (NOA-16)	1	39	IDH1 peptide vaccine	Safety, tolerability, and immunogenicity. Vaccine-induced immune response in 93%	NCT02454634 Completed
Epidermal Growth Factor Receptor Variant III (EGFRvIII)	Rindopepimut/Granulocyte Macrophage Colony Stimulating Factor (GM-CSF) In cases With Nd	3	745	TMZ vs. Rindopepimut + TMZ	OS = 20.1 (95% CI 18.5–22.1) vs. 20.0 m (95% CI 18.1–21.9)	NCT01480479 Completed
	GBM (ACTIVATe IV)	2	73	Rindopepimut + bevacizumab vs. Bevacizumab	PFS at 6 m = 28% vs. 16% (HR = 0.72, 95% CI 0.42–1.21)	NCT01498328 Completed
	Rindopepimut /GM-CSF in cases with EGFRvIII-Positive rGBM (ReACT)					
TERT	Anticancer Therapeutic by Telomerase-derived Universal Cancer Peptides vaccine in GBM (UCPVax-Glio)	1/2	56	UCPVax +TMZ vs. UCPVax	Anti-TERT T-cell response	NCT04280848 Active, not recruiting
	INO-5401 and INO-9012 vaccines delivered by EP and combined with Cemiplimab (REGN2810) in Nd GBM	1/2	52	MGMT INO-5401 + INO-9012 + Cemiplimab + RT + TMZ	mOS in MGMT-UN: 17.9 months vs. 32.5 months in MGMT-M	NCT03491683 Active, not recruiting

**Table 2:** Challenges and potential solutions.

Challenges	Potential Solutions
BBB and low infiltration of CAR-T cells in solid tumors [8,11,133,134,135,136,137]	Delivery of anticancer drugs across the BBB using nanoparticles (NCT00734682).
	Chemokine receptor expression in CAR-T cells [134,138,139,140,141].
	Loco-regional delivery of CAR-T cells [134,138,142,143,144,145].
Immunosuppressive TME [8,129,146,147,148,149,150,151,152,153,154,155,156,157,158]	CAR-T cells can release cytokines or antibodies inducing antibody-dependent cell-mediated cytotoxicity [149,159,160,161].
	Secretion of checkpoint-blocking antibodies by CAR-T cells or combination of CAR-T cell therapy with checkpoint inhibitors [156,162,163,164,165,166,167]. Elimination of co-inhibiting molecules [138,156,159,168,169,170,171,172]. Specific agonists targeting tumor-associated macrophages (TAM) [173,174,175,176]. Personalized vaccination therapy targeting neoantigens [175].
Intratumoral heterogeneity and antigen loss [105,133,159,177,178,179,180]	Multiple targeted and programmable CAR-T cells [181,182,183,184].
	Targeting cancer stem cells to avoid recurrence [8,149,185,186].
	Polytherapeutic approaches with radiotherapy or chemotherapy [148,187,188,189,190].
